# Bcl-3 regulates TGF*β* signaling by stabilizing Smad3 during breast cancer pulmonary metastasis

**DOI:** 10.1038/cddis.2016.405

**Published:** 2016-12-01

**Authors:** Xi Chen, Xinwei Cao, Xiaohua Sun, Rong Lei, Pengfei Chen, Yongxu Zhao, Yuhang Jiang, Jie Yin, Ran Chen, Deji Ye, Qi Wang, Zhanjie Liu, Sanhong Liu, Chunyan Cheng, Jie Mao, Yingyong Hou, Mingliang Wang, Ulrich Siebenlist, Y Eugene Chin, Ying Wang, Liu Cao, Guohong Hu, Xiaoren Zhang

**Affiliations:** 1 The Key Laboratory of Stem Cell Biology, Institute of Health Sciences, Shanghai Jiao Tong University School of Medicine (SJTUSM) & Shanghai Institutes for Biological Sciences (SIBS), Chinese Academy of Sciences (CAS), Shanghai 200025, China; 2 Department of Pathology, Zhongshan Hospital, Fudan University School of Medicine, Shanghai 200032, China; 3 Department of General Surgery, Ruijin Hospital, Shanghai Jiao-Tong University School of Medicine, Shanghai 200025, China; 4 Laboratory of Molecular Immunology, National Institute of Allergy and Infectious Diseases, National Institutes of Health, Bethesda, MD 20892, USA; 5 Collaborative Innovation Center of System Biomedicine, Shanghai Jiao Tong University School of Medicine, Shanghai 200240, China; 6 Liaoning Province Collaborative Innovation Center of Aging Related Disease Diagnosis and Treatment and Prevention, Shenyang 110001, China; 7 Key laboratory of Medical Cell Biology, China Medical University, Shenyang 110001, China

## Abstract

Transforming growth factor beta (TGF*β*) signaling in breast cancer is selectively associated with pulmonary metastasis. However, the underlying mechanisms remain unclear. Here we show that Bcl-3, a member of the IκB family, serves as a critical regulator in TGF*β* signaling to modulate breast cancer pulmonary metastasis. Bcl-3 expression was significantly associated with metastasis-free survival in breast cancer patients. Bcl-3 deletion inhibited the migration and invasion of breast cancer cells *in vitro*, as well as breast cancer lung metastasis *in vivo*. Bcl-3 was required for the expression of downstream TGF*β* signaling genes that are involved in breast cancer lung metastasis. Bcl-3 knockdown enhanced the degradation of Smad3 but not Smad2 following TGF*β* treatment. Bcl-3 could bind to Smad3 and prevent the ubiquitination and degradation of Smad3 protein. These results indicate that Bcl-3 serves as a promising target to prevent breast tumor lung metastasis.

Metastasis is the final and fatal stage of solid tumor progression and is responsible for most cancer-associated mortality. Transforming growth factor beta (TGF*β*) signaling has been implicated in many steps of metastasis and is positively associated with the distant metastasis of various types of cancers, including breast cancer.^1, 2, 3^ Studies based on mouse models of breast cancer have shown that TGF*β* signaling suppresses tumorigenesis but enhances the induction of epithelial–mesenchymal transition (EMT) and tumor invasion, consequently, promoting the seeding of lung metastases via genes like angiopoietin- like 4 (ANGPTL4).^3, 4, 5^ The activation of TGF*β* signaling results in the phosphorylation of transcription factors Smad2 and Smad3, which accumulate in the nucleus in association with Smad4 and transactivate downstream target genes.^6, 7^ Recent reports have shown that Smad2 and Smad3 may have different roles in cancer metastasis. In particular, Smad3 enhances metastasis, whereas Smad2 suppresses metastasis.^8, 9^ Importantly, the reversible phosphorylation and ubiquitination of Smad2 and Smad3 proteins are indispensable processes that regulate TGF*β* signaling.^10, 11^ Nedd4L has been reported to specifically recognize a TGF*β*-induced phospho-Thr-ProTyr motif in the linker region of Smad2/3 via the WW domain, which results in Smad2/3 polyubiquitination and degradation.^12^ OTUB1 inhibits the ubiquitination of phospho-Smad2/3 by binding to and inhibiting the E2 ubiquitin-conjugating enzymes, independent of its deubiquitinase activity.^13^ The RBX1-SCF*β*^-TrCP^ E3 ligase complex targets activated Smad3 for nuclear export and ubiquitin-mediated degradation upon its association with the transcriptional co-activator p300.^14^ However, the proteins that participate in the regulation of Smad3 degradation are largely unknown.

B-cell lymphoma 3 (*Bcl-3*) is a proto-oncogene in the region adjacent to the t(14; 19) (q32; q13) translocation that was first identified in a subset of patients with chronic lymphocytic leukemia (CLL).^15^ Unlike other members of the IκB family, Bcl-3 cannot inhibit NF-κB nuclear translocation but binds to p50 and p52 dimers on DNA and provides the complex with transactivation ability.^16, 17, 18^ Bcl-3 has important roles in innate and adaptive immunity.^19, 20^ In addition, Bcl-3 expression has been shown to increase in a variety of hematopoietic and solid cancers, including breast cancer, to regulate tumor development and progression.^21, 22, 23^ The protein Cylindromatosis (CYLD) binds and deubiquitinates Bcl-3, thereby prevents its nuclear accumulation.^26^ In cylindromas and many other cancers with reduced CYLD expression,^27^ increased nuclear accumulation of Bcl-3 induces the transcription of target genes, such as *cyclin d1* and *N-cadherin*, by interacting with NF-*κ*B p50 and p52 homodimers.^24, 25, 26, 27^ These data suggest that Bcl-3 may serve as an oncogene by regulating the cell cycle or apoptosis at the gene and protein levels during cancer development. We have showed that Bcl-3 stabilizes c-Myc protein and promotes colorectal cancer development by regulating ERK signaling.^22^ Bcl-3 was recently reported to promote colorectal tumorigenesis through activation of AKT signaling.^28^ The above evidence reveals that Bcl-3 has many novel roles in tumor development and progression beyond being a trans-activator or trans-repressor. Recently, Bcl-3 has been demonstrated to regulate the metastasis of mouse breast cancer cells.^21^ *Bcl-3* knockout MMTV-Neu mice resulted in reduced tumor lung metastasis with no effect on primary tumor growth.^21^ However, the underlying mechanisms of this metastasis remain unknown. Here we report that Bcl-3 functions as a critical regulator of TGF*β* signaling by stabilizing Smad3 to promote the pulmonary metastasis of breast cancer.

## Results

### Bcl-3 expression is associated with the metastasis of breast cancer

**Figure 1:**
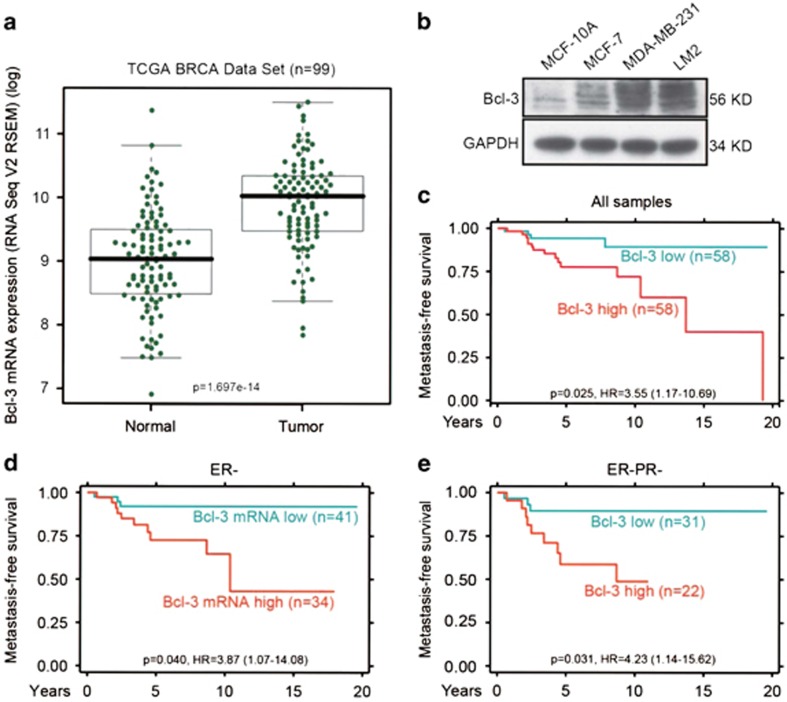
Bcl-3 expression is associated with the metastasis of breast cancer. (**a**) Expression of *Bcl-3* in human breast cancer clinical specimens using the TCGA BRCA dataset. (**b**) Western blot analysis of Bcl-3 in a panel of breast cancer cell lines. **(c)** The metastasis-free survival curve for breast cancer patients with low (*n*=58, blue curve) *versus* high (*n*=58, red curve) *Bcl-3* mRNA expression. (**d**) The metastasis-free survival curve for ER- breast cancer patients with low (*n*=41, blue curve) *versus* high (*n*=34, red curve) *Bcl-3* mRNA expression. (**e**) The metastasis-free survival curve for ER- PR- breast cancer patients with low (*n*=31, blue curve) *versus* high (*n*=22, red curve) *Bcl-3* mRNA expression. The patients were dichotomized on the basis of the mean value of *Bcl-3* mRNA expression, and their survival curves were later analyzed

We have previously reported that Bcl-3 was upregulated in human colorectal cancer compared with normal tissues.^22^ Bcl-3 expression has increased in breast cancers compared with normal mammary tissues.^29, 30^ Here we assessed *Bcl-3* mRNA expression levels based on the Cancer Genome Atlas (TCGA) breast cancer (BRCA) data^31^ and the expression of *Bcl-3* mRNA in tumors was much higher than tumor-matched normal tissues (*n*=99) (Figure 1a). Next, we detected the Bcl-3 expression of different breast cancer cell lines. Bcl-3 was highly expressed in malignant breast cancer cell lines but undetectable in MCF-10A, a normal mammary epithelial cell line (Figure 1b). Interestingly, the levels of Bcl-3 correlated with the pulmonary metastatic potential since highly metastatic cell lines LM2 expressed more Bcl-3 than cell lines with poor metastatic abilities like MCF-10A and MCF-7 (Figure 1b).These results suggest that Bcl-3 expression correlate with the pulmonary metastasis of breast cancer.

We then evaluated the elevated expression of *Bcl-3* with metastatic progression and metastasis-free survival in breast cancer patients. Patients with breast cancers (*n*=116, *P*=0.025) that expressed higher mRNA levels of *Bcl-3* had a significantly lower metastasis-free survival than patients whose tumors expressed lower levels of *Bcl-3* (Figure 1c). The same results were found in estrogen receptor negative (ER^−^) (*n*=75, *P*=0.040, Figure 1d) and ER^−^PR^−^ (progesterone receptors negative) (*n*=53, *P*=0.031, Figure 1e) breast cancers. These findings demonstrate a clinical significance of Bcl-3 in breast cancer and raise the need to further understand the function of Bcl-3 in breast tumor metastasis.

### Loss of Bcl-3 inhibits the pulmonary metastasis of breast cancer *in vivo*

**Figure 2:**
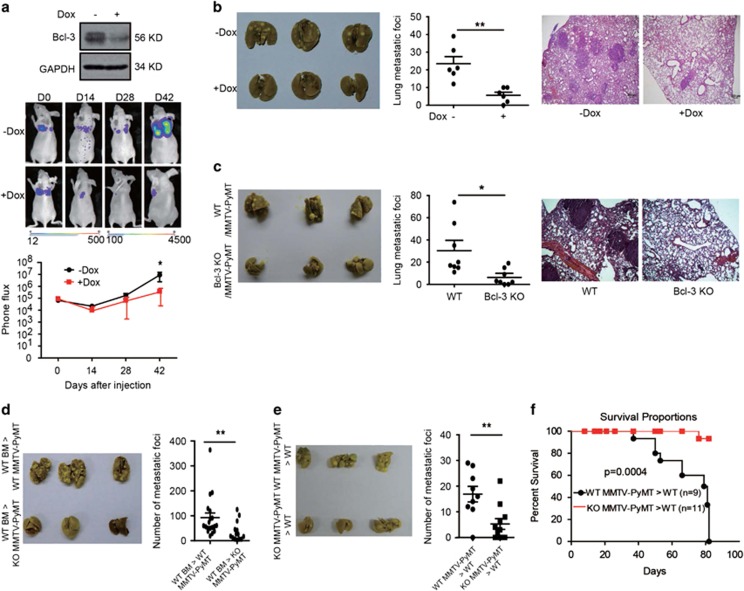
Loss of Bcl-3 inhibits the pulmonary metastasis of breast cancer *in vivo*. (**a**) Bcl-3 knockdown LM2 luciferase cells were injected into female nude mice through the tail vein. One group was fed normal water, the other received 2 *μ*g/ml Dox water. Representative lungs are shown by the BLI of the indicated cell lines at the indicated days after injection. Plots show a quantification of the luminescence signal as a function of time (*n*=6 for normal water; *n*=6 for Dox water). Values are mean±standard deviation. *P*<0.05 (two-tailed Student's *t-*test). (**b**) Lung metastases were counted 8 to 9 weeks after the inoculation of the indicated cells through the tail vain. Representative H&E sections and photos are shown. (**c**) The lungs of FVB/N-Tg (MMTV-PyVT) 634Mul/J (ID:002374) and *Bcl-3* knockout MMTV-PyVT female mice were harvested at necropsy and fixed with 4% polyoxymethylene; the number of lung metastasis foci were counted (*n*=8 for each group). Values are mean±S.D. *P*<0.05 (two-tailed Student's *t-*test). Representative H&E sections and photos are shown. (**d**) WT bone marrow cells were transferred into lethally irradiated *Bcl-3*-sufficient or *Bcl-3*-deficient MMTV-PyMT mice. The lungs were harvested at necropsy after fixation with 4% polyoxymethylene, and the lung metastasis foci were counted (*n*=19 for *Bcl-3*-sufficient MMTV-PyMT mice; *n*=14 for *Bcl-3*-deficient MMTV-PyMT mice). Values are mean±S.D. *P*<0.01 (two-tailed Student's *t-*test). (**e**) Cells from primary tumors of *Bcl-3*-sufficient or *Bcl-3*-deficient MMTV-PyMT mice were injected into the mammary pad of FVB mice. Lung metastases were counted 8 weeks after injection (*n*=9 for *Bcl-3*-sufficient MMTV-PyMT mice; *n*=11 for *Bcl-3*-deficient MMTV-PyMT mice). Values are mean±S.D. *P*<0.01 (two-tailed Student's *t-*test). (**f**) The survival curve for mice in (**e**)**.** *P<0.05 and **P<0.01 as determined by Student's *t*-test

To investigate the effects of Bcl-3 on the pulmonary metastasis of breast cancer *in vivo*, we depleted Bcl-3 in different breast cancer cell lines using an inducible Tet-on lentivirus system.^22^ After infection with lentivirus, the cells were exposed to doxycycline (Dox) (1 *μ*g/ml) for more than 4 days, and the expression of Bcl-3 was downregulated (Supplementary Figure 1c). Dox treatment did not affect the viability of cells (data not shown). We injected shRNA1-transduced LM2 cells, which were derived from parental MDA-MB-231 cells that exhibit highly pulmonary metastasis capability,^22, 32^ into nude mice via the tail vein, resulting in a substantial number of metastatic pulmonary nodules. The mice were fed with water containing Dox (2 *μ*g/ml) to induce Bcl-3 silencing. The pulmonary metastatic ability was assessed based on the luciferase activity on days 0, 14, 28 and 42 (Figure 2a). Dox-inducible Bcl-3 knockdown cells exhibited lower photon flux than the control group. The mice were sacrificed after 7 weeks, and an analysis of the lung tissues indicated that Bcl-3 knockdown reduced the number of metastatic pulmonary nodules (Figure 2b).

To confirm our observation in LM2 cells, we generated *Bcl-3*-sufficient and *Bcl-3*-deficient MMTV-PyMT mice, an aggressive transgenic mouse model of murine mammary adenocarcinoma development (MMTV-polyoma middle T (PyMT) mice).^33^ We found no gross histopathologic or quantitative differences between the groups based on primary tumor development or tumor burden (Supplementary Figure 2a). Strikingly, loss of Bcl-3 resulted in the attenuated pulmonary metastasis of breast cancer, as measured by significantly reduced numbers of metastatic foci and decreased tumor burden (Figure 2c). The pulmonary metastasis of mammary adenocarcinomas in MMTV-PyMT mice depends on hematopoietic cells, including CD4^+^ T cells, macrophages and TGF*β* signaling.^34^ To verify the role of Bcl-3 in the pulmonary metastasis of cancer cells, we transferred wild-type (WT) bone marrow into lethally irradiated *Bcl-3*-sufficient or *Bcl-3*-deficient MMTV-PyMT mice. We observed increased primary tumor volumes but significantly reduced numbers of lung metastatic foci in *Bcl-3*-deficient MMTV-PyMT mice (Supplementary Figure 2b and Figure 2d). In addition, we injected cells from primary tumors of *Bcl-3*-sufficient and *Bcl-3*-deficient MMTV-PyMT mice into the mammary gland of FVB mice. Comparable volumes of primary tumors from both *Bcl-3*-sufficient and *Bcl-3*-deficient breast cancer cells were observed (data not shown). However, the number of metastatic pulmonary nodules was significantly reduced in mice injected with cancer cells from *Bcl-3*-null MMTV-PyMT mice compared with WT MMTV-PyMT mice (Figure 2e).

Importantly, the reduced lung tumor burden in Bcl-3-ablated lungs translated into significantly prolonged survival after tumor initiation (Figure 2f). These results demonstrate that Bcl-3 promotes pulmonary metastasis but not tumorigenesis in breast cancer.

**Figure 3:**
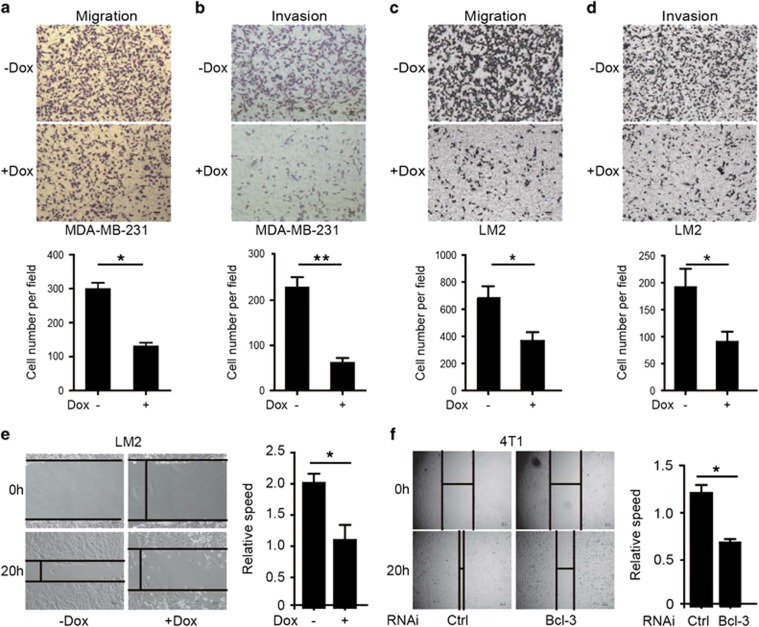
Loss of Bcl-3 inhibits the pulmonary metastasis of breast cancer cells. (**a–d**) Cell migration (**a, c**) and matrigel-transwell invasion (**b, d**) analysis of MDA-MB-231 cells (**a, b**) and LM2 cells (**c, d**), scale bar=50 *μ*m. **(e, f)** Wound-healing assay of Bcl-3 knockdown LM2 cells (**e**) and 4T1 cells (**f**), scale bar=50 *μ*m. Results are representative of three independent experiments. The data are expressed as mean±S.D. * represents *P*<0.05 and ** represents *P*<0.01 as determined by Student's *t*-test

To investigate the effects of Bcl-3 on pulmonary metastatic potential of breast cancer cells, we used *in vitro* systems to assess changes in cell motility and invasion. Knockdown of *Bcl-3* with two shRNA sequences in both MDA-MB-231 and LM2 cells led to significantly reduced migration (Figures 3a and c,Supplementary Figure 6c and d) and Matrigel invasion (Figures 3b and d, Supplementary Figure 6c and d) ability. Wound-healing assay showed that Bcl-3 depletion significantly reduced cell migration compared with control cells in LM2 and 4T1 cells (Figures 3e and f and Supplementary Figure 6b). Together, these results suggest that Bcl-3 promotes the pulmonary metastasis of breast cancer cells by regulating the migration and invasion of breast cancer cells.

### Bcl-3 regulates TGF*β* target gene expression

**Figure 4:**
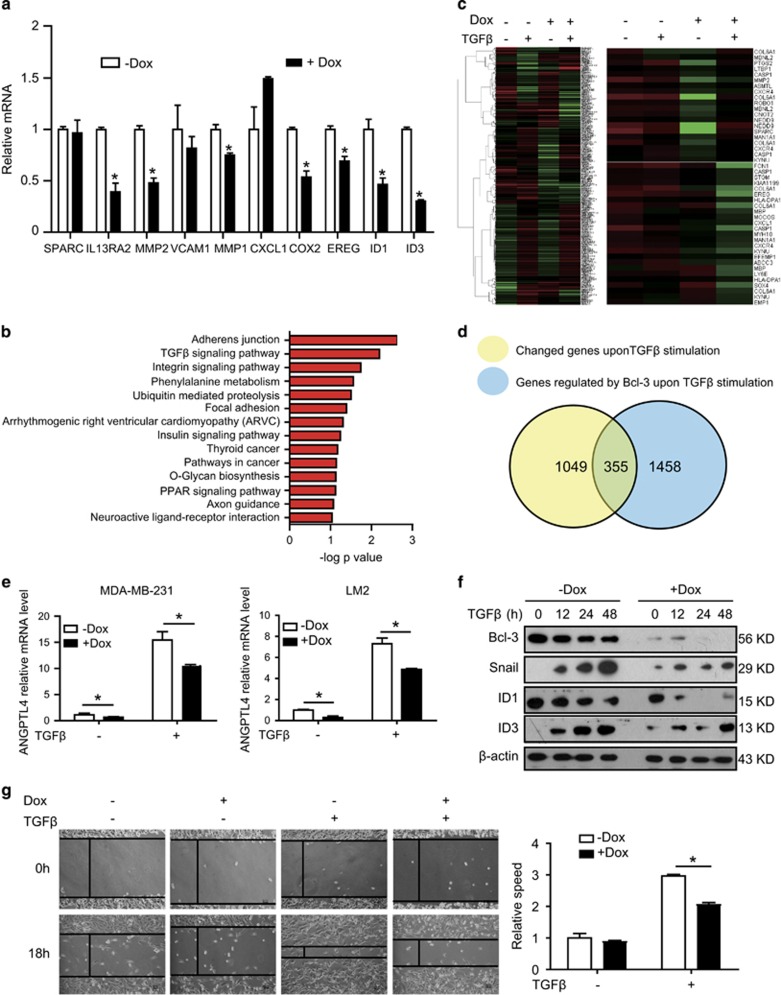
Bcl-3 regulates TGF*β* target gene expression. (**a**) qRT-PCR analysis of metastasis-related genes in Bcl-3 knockdown LM2 cells. (**b**) Gene ontology analysis shows altered signaling pathway. **(c)** Heat map of the altered genes shared between the four profiles; red shows induced and green shows repressed genes, log2-based scale. MDA-MB-231 cell RNA samples were collected after TGF*β* stimulation for 24 h. (**d**) Venn diagram representation of differentially expressed genes in the two groups: genes affected by TGF*β* stimulation and genes affected by Bcl-3 depletion after TGF*β* stimulation. (**e**) qRT-PCR analysis for *ANGPTL4* in the indicated MDA-MB-231 and LM2 cells with or without TGF*β* stimulation for 24 h. The results are the means±S.E.M.s for each cohort (*n*=3). Student's *t*-test was used. *P*<0.05. (**f**) Immunoblots for Bcl-3, Snail, ID1 and ID3 in the indicated MDA-MB-231 cells under TGF*β* stimulation. (**g**) Wound-healing assay carried out on the indicated MDA-MB-231 cells under TGF*β* stimulation, scale bar=50 *μ*m

To explore how Bcl-3 modulates the pulmonary metastasis of breast cancer, we detected a number of genes which are responsible for the enhanced pulmonary metastasis in LM2 cells, which has highly metastatic potential^35^ and autocrine production of TGF*β* (Supplementary Figure 3b). Bcl-3 deletion significantly reduced the expression of TGF*β* target genes *ID1* (inhibitor of DNA binding 1), *ID3* (inhibitor of DNA binding 1), *MMP1* (matrix metallopeptidase 1), and *COX2* (cytochrome c oxidase subunit II) in LM2 cells (Figure 4a). Next, globally profiled gene expression were performed to analyze genes affected by Bcl-3 depletion in MDA-MB-231 cells. Bcl-3 knockdown resulted in the differential expression of 1485 genes (>2-fold, *P*<0.05); 552 different genes were significantly upregulated (>2-fold increase, *P*<0.05), while 933 were significantly downregulated (<2-fold decrease, *P*<0.05) (Figure 4c). Gene ontology analysis showed that the most significantly reduced genes were associated with adherent junctions and the TGF*β* signaling pathway, suggesting that Bcl-3 might be involved in TGF*β* signaling (Figure 4b). To confirm that Bcl-3 was essential for TGF*β*-related gene expression, we compared gene expression patterns in Bcl-3-sufficient and Bcl-3-deficient MDA-MB-231 cells after 24 h TGF*β* stimulation. Bcl-3 knockdown caused a significant decrease in the expression of several TGF*β*-responsive genes and pulmonary metastasis-related genes (Figure 4c). Microarray analysis revealed that 1049 genes were affected by TGF*β* stimulation (428 induced and 621 repressed, >2-fold, Figure 4d), and knockdown of Bcl-3 after TGF*β* stimulation affected 1458 genes (396 induced and 1062 repressed, >2-fold, Figure 4d). In addition, gene expression profiles revealed an overlap (355 genes) between affected genes of TGF*β* stimulation and knockdown of Bcl-3 after TGF*β* stimulation (Figure 4d). TGF*β* primes LM2 cancer cells for pulmonary metastasis seeding via Angiopoietin-like potein 4 (ANGPTL4).^3^ The basal and TGF*β*-induced *ANGPTL4* mRNA levels were remarkably reduced in Bcl-3 depleted MDA-MB-231 and LM2 cells compared with control cells (Figure 4e). The functions of ID1 and ID3 are similar and redundant in regulating pulmonary metastasis,^36^ and Bcl-3 deletion reduced protein levels of both ID1 and ID3 (Figure 4f). Similarly, the TGF*β*-induced levels of *PAI-1*, *PTHrP*, *CTGF*, and *IL-11* (Supplementary Figure 3a) were dramatically decreased in Bcl-3-silenced MDA-MB-231 cells. ID1, ID3 and Snail, which are downstream targets of TGF*β* signaling,^37^ have been reported to enhance EMT and tumor metastasis.^36^ We observed the protein levels of ID3 and Snail induced by TGF*β* in Bcl-3-depleted MDA-MB-231 cells were profoundly reduced compared with the control cells. TGF*β* did not induce ID1 expression in MDA-MB-231 cells, TGF*β* instead reduced it when Bcl-3 was absent (Figure 4f). Consistently, Bcl-3 knockdown with shRNA1 sequence led to a significant reduction in wound healing induced by TGF*β* in MDA-MB-231 cells (Figure 4g). In LM2 cells not MDA-MB-231 cells, Bcl-3 knockdown led to the reduction in wound healing even without TGF*β* treatment (Figure 3e), the reason may be the higher autocrine production of TGF*β* in LM2 cells (Supplementary Figure 3b). Taken together, these results indicate that Bcl-3 promotes breast cancer metastasis and invasion by regulating TGF*β* signaling.

### Bcl-3 regulates TGF*β* signaling by stabilizing Smad3 protein

**Figure 5:**
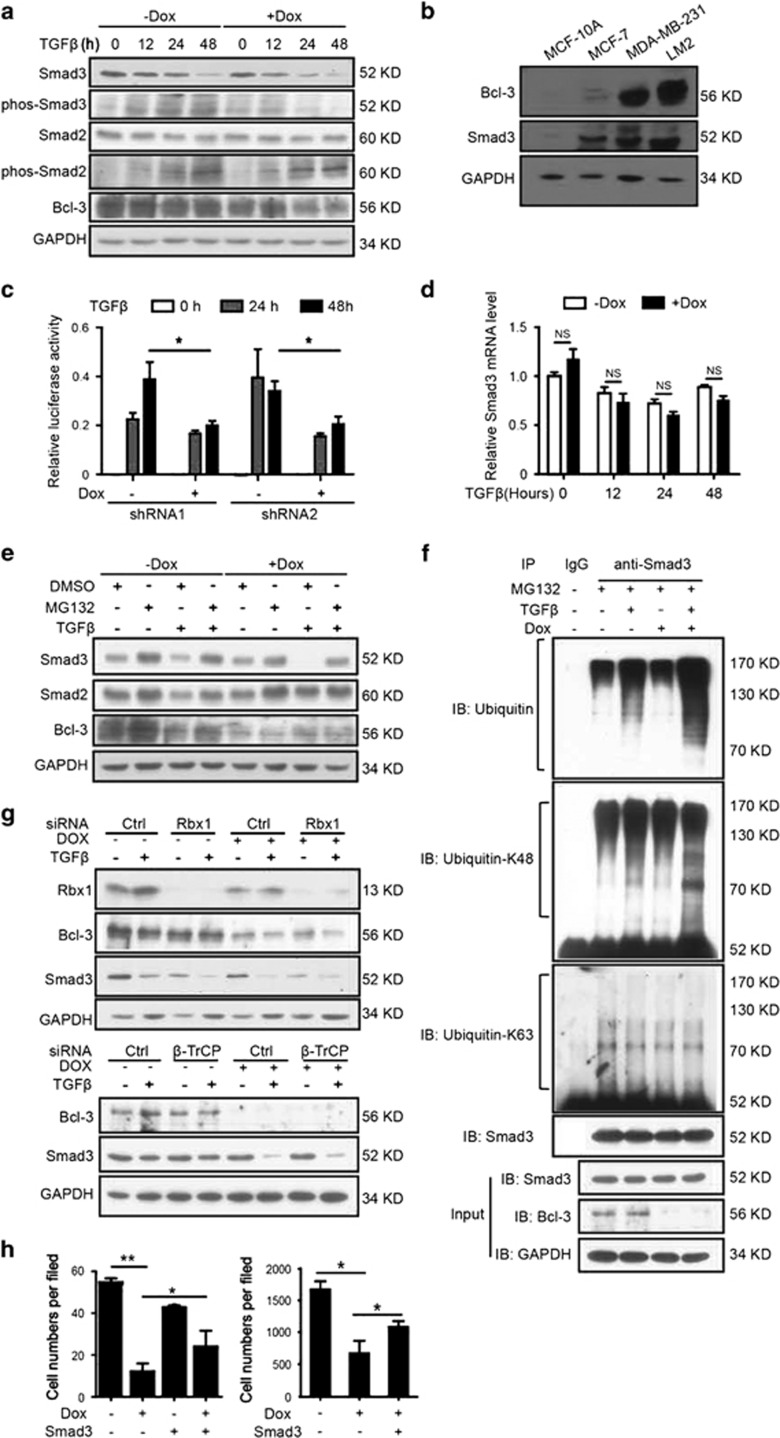
Bcl-3 regulates TGF*β* signaling by stabilizing Smad3 protein. (**a**) Immunoblots for Smad2, phospho-Smad2, Smad3 and phos-Smad3 in MDA-MB-231 cells under TGF*β* stimulation. (**b**) Immunoblots for Bcl-3 and Smad3 in MCF-10A, MCF-7, MDA-MB-231 and LM2 cells. (**c**) The knockdown of Bcl-3 inhibited the TGF*β* reporter luciferase activity in MDA-MB-231 cells. MDA-MB-231 cells with two different shRNA sequences were co-transfected with a TGF*β* reporter luciferase plasmid and a Renilla luciferase normalization control. After stimulation with TGF*β*, the cell lysates were collected, and the firefly luciferase and Renilla activities were measured with a dual-luciferase reporter system. The results are the means±S.E.M.s for each cohort. (*n*=3). *P<0.05. (**d**) qRT-PCR analysis of *Smad3* in Bcl-3 knockdown MDA-MB-231 cells treated with TGF*β*. (**e**) Immunoblots for Smad3 in Bcl-3 knockdown MDA-MB-231 cells treated with MG132 for 6 h and TGF*β*. (**f**) WT ubiquitination, K63-linked ubiquitination and K48-linked ubiquitination of Smad3 in MDA-MB-231 cells after MG132 and TGF*β* stimulation. The immunoblot analysis of immunoprecipitated Smad3 for the presence of ubiquitin, K48-linked ubiquitin and K63-linked ubiquitin, and Smad3 is shown. (**g**) Immunoblots for Smad3, Bcl-3 and GAPDH in RBX1 (up) or *β*-TrCP (bottom) knockdown MDA-MB-231 cells treated with TGF*β*. (**h**) Cell migration ability assessed by a transwell invasion analysis (left) and migration assay (right) in Smad3 overexpressing, Bcl-3-silenced MDA-MB-231 cells

To characterize how Bcl-3 regulates TGF*β* signaling, we examined the phosphorylation of Smad proteins in control and Bcl-3-silenced MDA-MB-231 cells. Bcl-3 knockdown did not influence the early phosphorylation and total levels of Smad2 or Smad4 induced by TGF*β*. Surprisingly, the phosphorylation and total protein levels of Smad3 decreased after TGF*β* treatment in Bcl-3 knockdown cells, and these changes were similar with ID1 protein expression (Figure 5a,Supplementary Figures 3c, d). Consistent results were obtained in Bcl-3-depleted LM2, MCF-7 cells and the mouse breast cancer cell line 4T1 (Supplementary Figures 3e-g). The results above indicate the correlation between Bcl-3 and Smad3. Then we considered the protein level of Bcl-3 and Smad3 in some breast cancer cells with different metastasis potential. Our results showed that these two proteins expressed consistently in these cells, as MCF-10A exhibited lowest Bcl-3 and Smad3 while LM2 cells showed the highest levels of Bcl-3 and Smad3 (Figure 5b). To explore whether Bcl-3 modulates Smad3-mediated gene transcription, we utilized a TGF*β* reporter plasmid^38^ to measure the TGF*β*-induced transcriptional activity. Knockdown of Bcl-3 with two shRNA sequences significantly inhibited the TGF*β* reporter luciferase activity induced by TGF*β* in MDA-MB-231 (Figure 5c) and MCF-7 (Supplementary Figure 3h) cells. These data indicate that Bcl-3 regulates TGF*β* signaling by affecting the protein levels of Smad3 but not Smad2 or Smad4.

The depletion of Bcl-3 in breast cancer cells reduced the steady-state levels of Smad3 protein upon TGF*β* stimulation but no change in the *Smad3* mRNA level (Figure 5d). These data suggest that Bcl-3 regulates the Smad3 levels via a posttranscriptional mechanism. Furthermore, the treatment of cells with the proteasome inhibitor MG132 restored the Smad3 protein levels decreased by Bcl-3 knockdown after TGF*β* treatment, confirming that Smad3 was degraded by the ubiquitin–proteasome system (Figure 5e). These data indicate that Bcl-3 depletion increases the proteasome-dependent degradation of Smad3 following TGF*β* stimulation. Indeed, Smad3 was found to be ubiquitinated in a K48-linked but not K63-linked manner in Bcl-3-depleted MDA-MB-231 cells after MG132 treatment and TGF*β* stimulation (Figure 5f and Supplementary Figure 3j).

An E3 ubiquitin ligase complex RBX1-SCF*β*^-TrCP^ is responsible for the ubiquitination of TGF*β*-activated Smad3.^14^ This E3 ligase complex is composed of RBX1 (RING box protein 1), Skp1, Cullin1 and *β*-TrCP.^14^ However, the depletion of RBX1 or *β*-TrCP could not lead to an accumulation of Smad3 in Bcl-3-sufficient or Bcl-3-silenced MDA-MB-231 cells following TGF*β* stimulation (Figure 5g). These results indicate that Bcl-3 stabilizes Smad3 via the ubiquitination dependent degradation pathway, but not regulated by the E3 ligase complex RBX1-SCF*β*^-TrCP^.

In addition, the significantly reduced invasion and wound-healing migration rates caused by the knockdown of Bcl-3 were partially rescued by Smad3 overexpression in Bcl-3-depleted LM2 cells (Figure 5h). Rescue experiments *in vitro* further confirm that Bcl-3-depletion induced decreases of Smad3 protein in breast cancer cells contribute to reduced migration and invasion ability *in vitro* and lung metastasis *in vivo*.

### Bcl-3 stabilizes Smad3 by forming a complex with Smad3

Phosphorylation at different sites of Smad3 contributes to its stability.^39^ To determine if Bcl-3 stabilized the Smad3 protein level via influencing Smad3 phosphorylation, we measured the phosphorylation levels at several Smad3 phosphorylation sites after TGF*β* stimulation in LM2 and MDA-MB-231 cells, including phosphor-Smad2/Smad3 Thr8, phosphor-Smad2/Smad3 Thr179, phosphor-Smad3 Ser204, phosphor-Smad3 Ser208, phosphor-Smad3 Ser213 and phosphor-Smad3 Ser423/425. The phosphorylation at Ser204, which is located in the linker region of Smad3, was significantly reduced in Bcl-3-depleted LM2 cells (Supplementary Figure 4a), but not in MDA-MB-231 (Supplementary Figure 4b). Moreover, we transfected Bcl-3-silenced MDA-MB-231 cells with WT Smad3, a Smad3 MH2 domain phosphorylation mutant called dSSVS, or a Smad3 linker region phosphorylation mutant called Smad3 EPSM. Similar to WT Smad3, decreased levels of mutated Smad3 proteins were detected in Bcl-3-depleted cells upon TGF*β* treatment (Supplementary Figure 4c). Taken together, these results indicate that the phosphorylation of Smad3 is not required for its regulation by Bcl-3 in TGF*β* signaling, even though Bcl-3 affected the Smad3 phosphorylation at Ser204 in LM2 cells.

**Figure 6:**
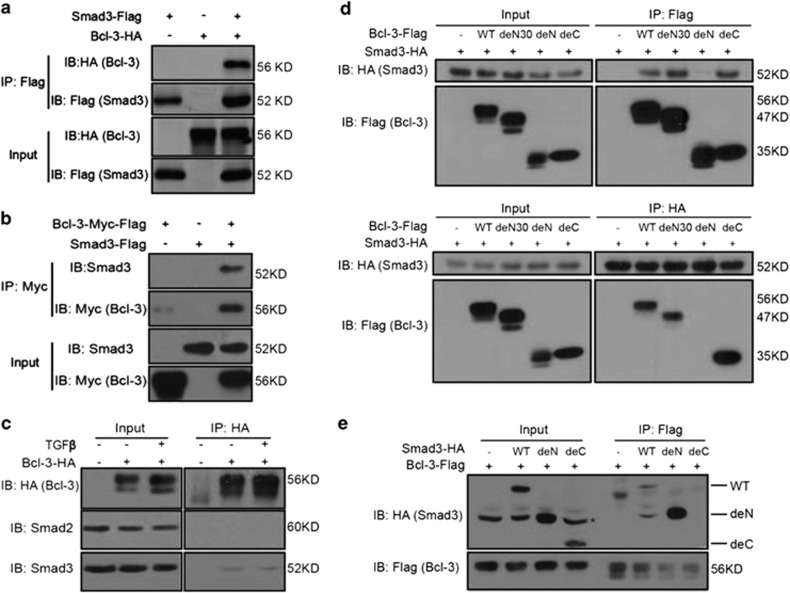
Bcl-3 stabilizes Smad3 by forming a complex with Smad3. (**a**) Co-IP of Flag-tagged Smad3 (Smad3-Flag) and hemagglutinin-tagged-Bcl-3 (Bcl-3-HA) expressed in HEK293T cells. (**b**) Co-IP of Flag-Myc-tagged Bcl-3 (Bcl-3-Flag-Myc) and Flag-tagged Smad3 (Smad3-Flag) expressed in HEK293T cells, cell lysates were immunoprecipitated with Myc antibodies. (**c**) HA-Bcl-3 was expressed in HEK293T cells. Forty-eight hours after transfection, the cells were lysed, and lysates were immunoprecipitated with HA antibodies. (**d**) Co-IP of Smad3-HA and Bcl-3 truncated expression vectors (WT: 1–446 amino acids; deN30: 31–446 amino acids; deN:125–446 amino acids; deC:1–330 amino acids) in HEK293T cells and cell lysates were immunoprecipitated with Flag (upper) and HA (bottom) antibodies. (**e**) Co-IP of Bcl-3-Flag and Smad3 truncated expression vectors (WT: 1–426 amino acids; deN: 138–426 amino acids; deC: 1–233 amino acids) in HEK293T cells and cell lysates with Flag antibodies. * represents nonspecific band. The deN mutant has the same molecular weight with nonspecific band

Given that Bcl-3 was selectively needed to stabilize Smad3 not Smad2, we hypothesized that Bcl-3 forms a complex with Smad3 to prevent the degradation of Smad3. First, we co-transfected expression vectors encoding Bcl-3 and Smad3 into 293 T cells and immunoprecipitated the lysates with antibodies directed against either protein tag. Immunoblots revealed that Bcl-3 was present in Smad3 immunoprecipitates and vice versa (Figures 6a and b). Further mapping revealed that an N-terminal deletion mutant of Bcl-3 (deN) could not interact with Smad3 (Figure 6d), while the C-terminal (MH2 domain) deletion (deC) mutant of Smad3 could not bind with Bcl-3 (Figure 6e). Consistently, Bcl-3 did not interact with endogenous Smad2 (Figure 6c). These results demonstrate that Bcl-3 binds to the MH2 domain of Smad3 to stabilize Smad3 by disrupting the degradation of Smad3.

Without TGF*β* treatment, Smad3 was located in the cytoplasm, whereas Bcl-3 were predominantly detected in the nucleus. Under TGF*β* stimulation, Smad3 trans-located into the nucleus where Bcl-3 co-localized with Smad3. When Bcl-3 was knocked down, the levels of Smad3 in the nucleus profoundly decreased after TGF*β* treatment. An immunofluorescence analysis revealed that Bcl-3 interacted with Smad3 in the nucleus, protecting Smad3 from degradation (Supplementary Figure 5).

## Discussion

TGF*β* signaling has a dual role in breast cancer tumorigenesis:^40, 41^ it inhibits tumor proliferation in the early stage, and promotes tumor metastasis via EMT in the advanced stages of carcinogenesis. Our study showed that Bcl-3 regulated the process of breast cancer pulmonary metastasis without affecting tumor proliferation. We found the elevated expression of Bcl-3 correlated with metastatic progression and metastasis-free survival in breast cancer patients. Moreover, Bcl-3-deficient MMTV-PyMT mice showed a significantly reduced number of pulmonary metastatic foci. Bcl-3 knockdown dramatically reduced the migration and invasion of breast cancer cells.

Bcl-3 was recently shown to be able to promote the metastasis of ErbB2-positive mammary tumors,^21^ but little is known about the mechanism. In our study, we found that Bcl-3 regulated breast cancer pulmonary metastasis in breast cancer cells by modulating TGF*β* signaling via the stabilization of Smad3 and other metastasis-related proteins such as ID1 (see discussion below).

Researchers have investigated the specific functions of Smad2 and Smad3 in TGF*β*-induced signaling in breast cancer cells *in vitro* and in a mouse model of breast cancer metastasis. The knockdown of Smad3 in MDA-MB-231 cells resulted in the prolonged latency and delayed growth of metastases, whereas Smad2 knockdown resulted in a more aggressive phenotype compared with control cells.^9^ Therefore, we demonstrated that Bcl-3 serves as a critical regulator in stabilizing Smad3, but not Smad2 or Smad4, upon TGF*β* stimulation. This function is crucial for the TGF*β*-dependent cell migration, invasion and pulmonary metastasis of breast cancer.

Notably, Smad3 overexpression only partially rescued the deficiency caused by Bcl-3 loss, suggesting that other mechanisms might be involved in the regulation of the pulmonary metastasis of breast cancer by Bcl-3. First, Bcl-3 might regulate EMT independently of Smad3. Indeed, we found that Bcl-3 depletion in some breast cancer cell lines, such as MDA-MD-231 and 4T1 cells, led to mesenchymal-to-epithelial transition (MET) induction, there was as demonstrated by reduced F-actin polarity (Supplementary Figure 4d) and an obvious morph-change from fibroblast-like to round-like (Supplementary Figure 4e). Second, Bcl-3 may regulate the stability of other metastasis-related proteins in addition to Smad3. For example, we observed that the protein levels of ID1 were regulated by Bcl-3, especially upon TGF*β* treatment. However, we did not detect the interaction of Bcl-3 with these proteins. The mechanisms underlying the Bcl-3 regulation of metastasis need to be further investigated.

TGF*β* signaling is mainly determined by the expression level and activity of the effector proteins Smad2 and Smad3.^39^ After TGF*β* stimulation, Smad3 can be recognized by the E3 ligase RBX1 at its MH2 domain and then degraded by the ubiquitin–proteasome pathway.^14^ Smad3 is phosphorylated at Ser208 by ERK MAP kinase upon EGF treatment, and ERK phosphorylation inhibits Smad3 activity because the mutation of the ERK phosphorylation sites increases the ability of Smad3 to stimulate a Smad target gene.^42^ We found that the phosphorylation at Ser204, located in the link region of Smad3, was significantly reduced in Bcl-3-depleted cells, which might be accounted for by our previous finding that Bcl-3 depletion reduced ERK activation^22^ (data not shown). However, the Smad3 linker region phosphorylation did not differ between mutant EPSM and control cells after TGF*β* treatment. These results indicate that the stabilization of Smad3 by Bcl-3 does not depend on the phosphorylation of the Smad3 linker domain.

Importantly, Bcl-3 directly interacts with the MH2 domain of Smad3, which is consistent with reports that DEAR1 and AKT bind to the MH2 domain of Smad3 but do not interact with Smad2.^43, 44, 45^ MH2 domain of Smad3 is the binding site of E3 ligase or adapter such as DEAR1 that promote Smad3 ubiquitination and degradation. The binding of Bcl-3 with MH2 domain of Smad3 but not Smad2, suggest that Bcl-3 stabilizes Smad3 through competitively preventing the interaction of Smad3 with its E3 ligase, probably other proteins such as DEAR1. Therefore, we propose that Bcl-3 stabilizes Smad3, but not Smad2 or Smad4, by disrupting the interaction of Smad3 and its E3 ligase, preventing the degradation of Smad3 by the ubiquitin–proteasome pathway. However, the depletion of RBX1 or *β*-TrCP, the components of the E3 ubiquitin ligase complex which is responsible for the ubiquitination of TGF*β*-activated Smad3, could not lead to an accumulation of Smad3 in MDA-MB-231 cells whether TGF*β* stimulation or not, maybe other E3 ubiquitin ligase participate in this process. This process is not associated with the phosphorylation of Smad3, but depend on the binding affinity of Bcl-3 and Smad3.

In summary, our study uncovered a novel mechanism by which Bcl-3 stabilizes Smad3 protein during TGF*β* signaling. Because TGF*β* signaling plays important roles in breast cancer pulmonary metastasis,^46^ our findings provide a promising new drug target for the prevention and therapy of breast cancer pulmonary metastases.

## Materials and Methods

### Cell lines

The human breast cancer cell lines HEK293T, MDA-MB-231, MCF-10A, MCF-7, were purchased from ATCC (Rockville, MD, USA). LM2 was a kind gift from Dr Joan Massagué. The mouse breast cancer cell line 4T1 was purchased from SGST (Shanghai, China). HEK293T, MDA-MB-231, LM2 and MCF-7 were cultured in Dulbecco's Modified Eagle's Medium (DMEM, HyClone, Logan, UT, USA) supplemented with 10% FBS, 1% penicillin and streptomycin in a 5% CO_2_ humidified atmosphere at 37 °C. 4T1 cells were maintained in RMPI-1640 medium (HyClone, Logan, UT, USA) supplemented with 10% FBS, 100 units/ml penicillin and 100 *μ*g/ml streptomycin.

### Patient data

Breast cancer tissues were collected from patients at Qilu Hospital of Shandong University. All of these tumors were histologically confirmed as breast carcinomas. Informed consent for participation in this study was obtained from all patients before their surgeries. And human subject studies were approved by the institutional review boards of Qilu Hospital with informed patient consent.

### Lentivirus-delivered shRNA gene knockdown and overexpression

To knockdown the *Bcl-3* gene in breast carcinoma cell lines, two different Bcl-3 shRNA sequences were cloned into the pTRIPZ plasmid (Open Biosystems, RHS4750, Huntsville, Alabama, USA), a tet-on lentiviral vector, according to the manufacturer's instructions (V3THS_407972), respectively. The third shRNA sequence targeting Bcl-3 was cloned into plvx-shRNA plasmid. The non-silencing lentiviral shRNA vector was used as a control. The lentivirus were packaged using psPAX2 and pMD2G, a three-plasmid system. To obtain stable cell lines, lentivirus supernatant was added to MDA-MB-231, LM2 and 4T1 cells, followed by screening with 1 *μ*g/ml puromycin for MDA-MB-231, 0.5 *μ*g/ml puromycin for MCF-7, and 14 *μ*g/ml puromycin for 4T1 cells for 2 weeks. The expression of Bcl-3 was downregulated in these cell lines when treated for longer than 4 days with 1 *μ*g/ml doxycycline (Dox), an analog of tetracycline, in the culture medium. To overexpress Smad3 in Bcl-3 knockdown cells, Smad3 was cloned into the pLVX-IRES-ZsGeen1 plasmid. Lentivirus supernatant was added to the culture medium of Bcl-3 knockdown cells. We assessed the infection rate using a fluorescence microscope.

### TCGA data analysis

The RNA-seq dataset were retrieved from the Cancer Genome Atlas (TCGA) database and only appropriate tumor-normal matched samples were included (sample size=99). RSEM normalized results from TCGA were applied to downstream analysis, and we manually checked expression level of Bcl-3 in these samples.

### Plasmids and antibodies

Flag-tagged Bcl-3, or HA-tagged Smad3 were cloned into pcDNA3.1 and pcDNA3.0. All PCR products were confirmed by sequencing. The following antibodies were used: anti-Bcl-3, c-Myc, cyclinD1, p27, p21, vimentin, E-cadherin, N-cadherin, ID1, FLAG, HA, ubiquitin (Santa Cruz Biotechnology, Inc., Santa Cruz, CA, USA), anti-Snail, ID3, RBX1 (Proteintech), anti-phos-ERK, phos-AKT, AKT, phos-Smad3, Smad3, phos-Smad2, Smad2, Lamin A/C (Cell Signaling Technology, Boston, MA, USA), anti-GAPDH, donkey anti-Goat IgG (HRP), goat anti-Mouse IgG (HRP), goat anti-Rabbit IgG (HRP) (KANGCHEN, Shanghai, China), anti-Actin (Sigma-Aldrich, St. Louis, MO, USA), anti-K63-ubiquitin, K48-ubiquitin (Millipore, Billerica, MA, USA), donkey anti-goat coupled to AlexaFluor®488, and donkey anti-mouse or rabbit IgG coupled to AlexaFluor680 (Invitrogen, Carlsbad, CA, USA). Anti-Smad3 Ser423/425 (Cell Signaling Technology), anti-Smad2/3 pT8, anti-Smad2/3 pT179, anti-Smad3 pS204, anti-Smad3 pS208, anti-Smad3 pS213 were kind gifts from Dr Liu Fang.

### Immunoprecipitation and immunoblotting

Confluent cells in 10-cm tissue culture dishes were washed with PBS containing 10 mM CaCl_2_ and then lysed in 1 ml of lysis buffer (20 mM Hepes (pH 7.4), 12.5 mM *β*-glycerophosphate, 0.5% Triton-X-100, 150 mM NaCl, 1.5 mM MgCl, 2 mM EGTA, supplemented with phosphatase and protease inhibitors) and incubated on ice for 30 min before clearing by centrifugation at 14 000 r.p.m. for 15 min. The proteins from cell extracts were immunoprecipitated out using protein A-Sepharose (GE, Fairfield, CT, USA) coated with specific antibodies. Following overnight incubation at 4 °C, immunocomplexes were collected and washed four times with lysis buffer. Bound proteins were eluted with SDS sample buffer and analyzed by SDS-PAGE. Immunoblotting was carried out using standard procedures, and immune-reactive proteins were visualized by enhanced chemiluminescence (ECL). For ubiquitination assay, MG-132 (Beyotime, Shanghai, China s1748, 100 mmol/l for 12 h) were used to inhibit proteasome activity.

### Mice

All animals were housed and maintained in pathogen-free conditions. All animal experiments were performed in compliance with the guide for the care and use of laboratory animals and were approved by the institutional biomedical research ethics committee of the Shanghai Institutes for Biological Sciences at the Chinese Academy of Sciences. Female BABL/c nude mice were purchased from the Shanghai Laboratory Animal Center at the Chinese Academy of Sciences in Shanghai. The Bcl-3 knockout (KO) mice have been described previously.^47^ Female Bcl-3^−/−^ mice were crossed with male FVB mice that were transgenic (+/−) for the PyMT antigen under the control of the MMTV promoter. Genotyping for the PyMT transgene was performed by PCR. Female mice from this cross that were PyMT^+/−^ were saved for further analysis. The mice were sacrificed at 15 weeks of age, and the whole mammary glands, tumors and/or lungs were excised.

### Histology and immunohistochemistry

Anti-Bcl-3 antibodies (Santa Cruz Biotechnology, Inc. Santa Cruz, CA, USA) were used as the primary antibodies. IHC analyses of diaminobenzidine staining were performed using an HRP kit (UltraTek; Scytek, Logan, UT, USA). The diaminobenzidine-stained specimens were visualized using a general optical microscope with a camera (Carl Zeiss, Oberkochen, Germany). Hematoxylin and eosin (H&E) staining of the tissue was conducted using a MIRAX scan (Carl Zeiss). Images were processed with equivalent parameters using ZEN Light Edition software (Carl Zeiss).

### Immunofluorescence

Immunofluorescence was analyzed as described previously.^48^ The cells were plated at 3 × 10^5^ cells per 35 mm dish onto coverslips and washed 3 times in 100 mM PBS before being fixed in cold acetone (5 min, room temperature). The cells were blocked with PBS containing 1% FBS and 0.3% Triton-X-100 for 30 min. The proteins were localized using the primary antibodies overnight at 4 °C. The cells were washed and incubated with secondary antibodies coupled to AlexaFluor®488 or AlexaFluor®680 (1:500, 2 h, room temperature). The cells were then observed with a Zeiss laser-scanning confocal microscope (LSM Meta 510) using a Fluar Plan Apochromat × 63 oil immersion objective (numerical aperture 1.4) or a Plan Apochromat × 100 oil immersion objective (numerical aperture 1.4). The images were collected at a zoom of 2–3 and an iris of <3 *μ*m. Single sections are shown. The images were processed (colored and merged) using the Zeiss (LSM 510) software. More than 20 cells were analyzed for each experiment.

### Luciferase assay

TGF*β* reporter luciferase plasmid^38^ was provided by Dr Ying E Zhang of NCI. MDA-MB-231 cells were cultured in 24-well dishes and transfected with 50 ng of TGF*β* reporter luciferase plasmids and 20 ng of Renilla plasmids using Lipofectamine 2000. After 24 h of transfection, the cells were lysed in reporter lysis buffer (Promega, Madison, WI, USA). The cell extracts were collected, and the firefly luciferase and Renilla activities were measured with a Dual-Luciferase Reporter System (Promega).

### RNA extraction and real-time PCR

RNA was isolated from cell lines or patient samples using TRIzol Reagent (Invitrogen, 15596-018) according to the manufacturer's protocol. To obtain cDNA, reverse transcription was performed using Transcript First Strand Synthesis Supermix (TransGen Biotech, Beijing, China AT301) according to the manufacturer's instructions, using 1 *μ*g of RNA as a template. All qRT-PCRs were performed using a 7500 Fast Real-Time PCR System (Applied Biosystems, Carlsbad, CA, USA), and all qRT-PCR reagents and consumables were purchased from Applied Biosystems. For each reaction, 1 *μ*l of RT product was added to 10 *μ*l of 2X SYBR Green Gene Expression PCR Master Mix and 1 *μ*l of pre-designed and synthesized forward and reverse primer/probe mix. Each sample was analyzed in triplicate. Relative quantification (RQ) was derived from the difference in the cycle threshold (Ct) between the target gene and GAPDH (ΔCt) compared with control cell lines using the equation RQ=2^−ΔΔCt^. Error bars represent the standard deviation (SD), and the significance of differences was calculated using a one-tailed, unpaired *t*-test. The sequences of the primers are listed in Supplementary Table 1. The patients were dichotomized on the basis of the mean value of Bcl-3 mRNA expression, and their survival curves were later analyzed.

### Wound-healing assay

Cell monolayer in 6-well plates was artificially scratched with 10 *μ*l pipette tips. The wound areas were photographed 0 and 20 h after scratching and measured using a caliper. The wound-closure percentages were calculated using the following formula: (1-(current wound size/initial wound size)) × 100.

### *In vitro* invasion assays

*In vitro* invasion assays were conducted using Transwell inserts (Costar, Cambridge, MA, USA) containing 8-*μ*m pore-size polycarbonate membrane filters in 24-well culture plates. The upper surface of the filter was coated with 20 *μ*g of Matrigel (Becton Dickinson, Bedford, MA, USA) per filter. The Matrigel was dried and reconstituted at 37 °C into a solid gel on the filter surface. After starving in FBS-free DMEM overnight, 1 × 10^5^ MDA-MB-231 cells were seeded in the upper chamber in 200 *μ*l of 0.1% BSA DMEM. The lower chamber was flooded with 500 *μ*l of 10% FBS DMEM. After 18 h, the wells were fixed with 4% polyoxymethylene and stained with crystal violet. Cells that invaded the lower surface of the filter were counted in 6 random fields under a light microscope at high magnification. Experiments were repeated at least in triplicate.

### *In vivo* metastasis assays

For experimental metastasis assays, age-matched female nude mice were injected with 2 × 10^5^ LM2 cells stably expressing an inducible Bcl-3 knockdown vector in PBS via the tail vein. After 7 weeks, the mice were sacrificed, and the lungs were collected and immobilized in Bouin's solution to count the number of foci and stain the tissues with hematoxylin and eosin (H&E). To observe living bodies, 2 × 10^5^ LM2 with the indicated constructs were injected into age-matched female nude mice via the tail vein, and D-luciferin was retro-orbitally injected at days 1, 14, 28 and 42. The photon flux (photons per second) was recorded.

### Gene expression profiling

The total RNA was extracted using TRIzol Reagent (Cat No.15596-018, Life technologies, Carlsbad, CA, USA) following the manufacturer's instructions and checked for a RIN number to inspect RNA integrity with an Agil manufacturer's ent Bioanalyzer 2100 (Agilent technologies, Santa Clara, CA, USA). Qualified total RNA was further purified with the RNeasy micro kit (Cat No. 74004, QIAGEN, GmBH, Germany) and RNase-Free DNase Set (Cat No. 79254, QIAGEN, GmBH, Germany). The total RNA was amplified, labeled and purified using a GeneChip 3'IVT Express Kit (Cat No. 901229, Affymetrix, Santa Clara, CA, USA) following the manufacturer's instructions to obtain biotin-labeled cRNA. Array hybridization and washing was performed using a GeneChip® Hybridization, Wash and Stain Kit (Cat No. 900720, Affymetrix, Santa Clara, CA, USA) in a hybridization oven 645 (Cat No. 00-0331-220 V, Affymetrix, Santa Clara, CA, USA) and a fluidics station 450 (Cat No. 00-0079, Affymetrix, Santa Clara, CA, USA) following the manufacturer's instructions. The slides were scanned by a GeneChip Scanner 3000 (Cat No. 00-00212, Affymetrix, Santa Clara, CA, USA) using the Command Console Software 3.1 (Affymetrix, Santa Clara, CA, USA) and default settings. The raw data were normalized using the MAS 5.0 algorithm, Gene Spring Software 11.0 (Agilent technologies, Santa Clara, CA, USA). For the gene ontology analyses, probe IDs were converted to unigene IDs, and unigenes were assigned gene ontology terms from the gene ontology database based on the closest gene ontology-annotated BLASTX homolog.

### Primary breast cancer cell isolation from MMTV- PyMT mice

The tissues were minced into small (1–2 mm) pieces and digested with 5% FBS in DMEM containing 2 mg/ml collagenase I and 2 mg/ml hyaluronidase (Sigma-Aldrich) at 37 °C for 2 h. The cells were sequentially filtered through a 70-μm cell strainer and washed with PBS. The cells were then centrifuged in a Beckman Allegra X-15 R centrifuge at 300 × *g* for 10 min. The harvested cells were cultured in DMEM.

### Mouse bone marrow transfer assay

Female FVB-MMTV-PyMT and *Bcl-3*^−/−^ MMTV-PyMT mice (6–8 weeks old) were subjected to 8.5 Gy total body irradiation before being injected with 5 × 10^6^ bone marrow cells isolated from female FVB mice in 0.2 ml of medium via the tail vein.

### Statistical analyses

Unless stated otherwise, all experiments were repeated three times and all data were presented as mean±standard deviation. All other experiments were analyzed using two-tailed Student's *t*-tests. *P*⩽0.05 was considered statistically significant.

## Supplementary Material

Supplementary Materials-red Highlight
