# Supplementary material for: Bcl-3 regulates TGFβ signaling by stabilizing Smad3 during breast cancer pulmonary metastasis
Source: Cell Death Dis. 2016 Dec 1;7(12):e2508–. doi: 10.1038/cddis.2016.405 (PMC5261001; doi:10.1038/cddis.2016.405)
Supplement: Supplementary Materials [file cddis2016405x2.pdf]

Supplementary figures

Supplementary Figure 1 Bcl-3 knockdown strategies

(**a**) Bcl-3 knockdown strategy, an inducible lentivirus pTRIPZ plasmid invented by OpenBiosystems was used.

(**b**) Two shRNA sequences for human Bcl-3 knockdown. White indicates the enzyme, red and blue indicate the knockdown sequence and its reverse complementary sequence. Green indicates the loop.

(**c**) The effect of Bcl-3 knockdown with two shRNA sequences in different breast cancer cells.

Supplementary Figure 2 Tumor volume of WT and Bcl-3 knockout MMTV-PyMT mice

(**a**) Tumor volume of Bcl-3-sufficient or Bcl-3-deficient MMTV-PyMT mice. The tumor burden had no changes between the groups after 3.5 months.

(**b**) WT bone marrow cells were transferred into lethally irradiated Bcl-3-sufficient or Bcl-3-deficient MMTV-PyMT mice. Tumor volumes were measured.

Supplementary Figure 3 Bcl-3 regulates TGFβ signaling by stabilizing Smad3 protein

(**a**) qRT-PCR analysis of metastasis-related genes in Bcl-3 knockdown MDA-MB-231 cells after TGFβ stimulation.

(**b**) qRT-PCR analysis of TGFβ-1 expression in MDA-MB-231 and LM2 cells.

(**c, d**) Immunoblots for Smad4 in MDA-MB-231 (**c**) and MCF-7 (**d**) cells under TGFβ stimulation.

(**e, f**) Immunoblots for Smad2, phospho-Smad2, Smad3 and phos-Smad3 of LM2 (**e**) and MCF-7 (**f**) cells under TGFβ stimulation.

(**g**) Immunoblots for Smad3 and Smad2 of 4T1 cells after RNAi Bcl-3 under TGFβ stimulation.

(**h**) Luciferase activity of MCF-7 cells transfected with Smad3 luciferase-reporter plasmids and Renilla luciferase plasmids after TGFβ stimulation.

(**i**) qRT-PCR analysis of BTRC (β-TrCP) in BTRC knockdown MDA-MB-231 cells.

(**j**) Immunoblots for Smad3 in MDA-MB-231 cells after MG132 and TGFβ stimulation to detect the ubiquitinated Smad3.

Supplementary Figure 4 Bcl-3 regulation of TGFβ signaling is not correlated with the phosphorylation of Smad3

(**a and b**) Immunoblots for phospho-Smad3 with different phospho-antibodies in LM2 cell (**a**) and MDA-MB-231 cell (**b**) under TGFβ stimulation or untreated control cells.

(**c**) Immunoblots for Bcl-3 and Flag tagged Smad3 mutants in MDA-MB-231 cells after TGFβ stimulation.

(**d**) Fluorescence microscopy analysis of F-actin expression in MDA-MB-231 cells.

(**e**) Morph-Change of 4T1 cell after Bcl-3 was knocked down.

Supplementary Figure 5 Cellular localization of Bcl-3 and Smad3 in TGFβ treated or untreated MDA-MB-231 cells

Fluorescence microscopy analysis of Bcl-3 and Smad3 expression in MDA-MB-231 cells treated with TGFβ or untreated control cells. Blue, nucleus stain.

Supplementary Figure 6 Loss of Bcl-3 inhibits the metastasis of breast cancer

cells

(**a**) The knockdown effect of Bcl-3 in MDA-MB-231 and LM2 cells detected by Western Blot.

(**b**) Wound-healing assay in Bcl-3 knockdown LM2 cells.

(**c,d**) Cell migration and Matrigel-Transwell invasion analysis of MDA-MB-231 cells (**c**) and LM2 cells (**d**), scale bar = 50μm. * represents $p < 0.05$ and ** represents $p < 0.01$ as determined by Student’s *t*-test.

Q-RT-PCR primers (5’-3’)

TGF-β-1-F	GGCCAGATCCTGTCCAAGC
TGF-β-1-R	GTGGGTTTCCACCATTAGCAC
GAPDH-F	TGCACCACCAACTGCTTAGC
GAPDH-R	GGCATGGACTGTGGTCATGAG
ANGPTL4-F	TCTCCGTACCCTTCTCCACT
ANGPTL4-R	AGTACTGGCCGTTGAGGTTG
CTGF-F	TTGCGAAGCTGACCTGGAAGAGAA
CTGF-R	AGCTCGGTATGTCTTCATGCTGGT
PAI-1-F	TCTTTGGTGAAGGGTCTGCT
PAI-1-R	CTGGGTTTCTCCTCCTGTTG
IL-11-F	ACTGCTGCTGCTGAAGACTC
IL-11-R	CCACCCCTGCTCCTGAAATA
PTHrP-F	ACCTCGGAGGTGTCCCCTAAC
PTHrP-R	TCAGACCCAAATCGGACG
ID1-F	GGCTGTTACTCACGCCTCAAG
ID1-R	CCAACTGAAGGTCCCTGATGTAG
COX2-F	ACA ACA TTC CTT CCT TC
COX2-R	CCTTATTTCCTTTCACACC
ID3-F	TGAGCTTGCTGGACGACATG
ID3-R	GATGACGCGCTGTAGGATTTC
MMP1-F	GAGCAAACACATCTGACCTACAGGA
MMP1-R	TTGTCCCGATGATCTCCCCTGACA
BTRC-F	ACCAACATGGGCACATAAACTC
BTRC-R	TGGCATCCAGGTATGACAGAAT

shBcl-3-1 (5’-3’):
GAGCTCGAACCAACCTAAAGAAAACATTATCACTTCGGTGTCTACATAATGTTTTCTTTAGGTTGGTTTCTTAAG

shBcl-3-2 (5’-3’):
GAGCTCTGGCCCTCGAGCTGTAGATGTTATCACTTCGGTGTCTACATAACATCTACAGCTCGAGGGCCG CTTAAG

shBcl-3-3(5’-3’):
GATCCGTCGACGCAGTGGACATTAATTCAAGAGATTAATGTCCACTGCGTCGATTTTTTG

RBX1 siRNA: UCCAUAAUGUGGUUCCUGC
BTRC siRNA: AAGUGGAAUUUGUGGAACAUC
