# Supplementary material for: Bcl-3 regulates TGFβ signaling by stabilizing Smad3 during breast cancer pulmonary metastasis
Source: Cell Death Dis. 2016 Dec 1;7(12):e2508–. doi: 10.1038/cddis.2016.405 (PMC5261001; doi:10.1038/cddis.2016.405)

Supplementary Figure 1 Bcl-3 knockdown strategy

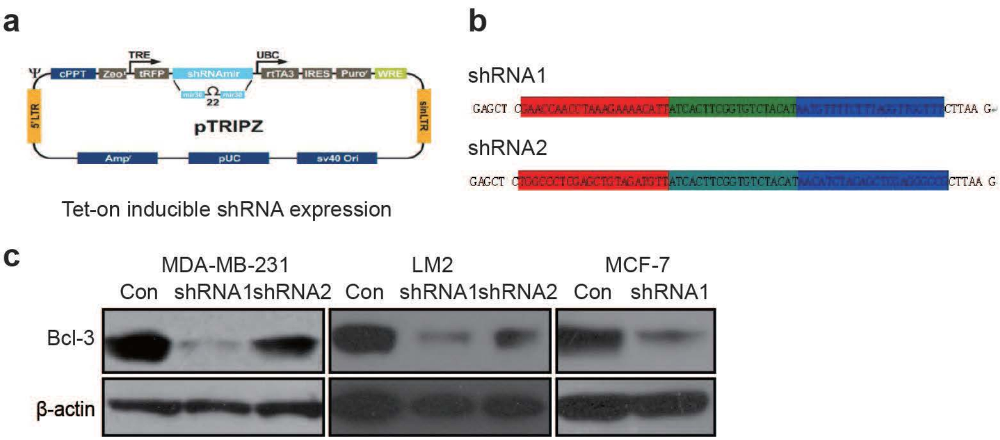

Supplementary Figure 2 Tumor volume of WT and Bcl-3 knockout MMTV-PyMT mice

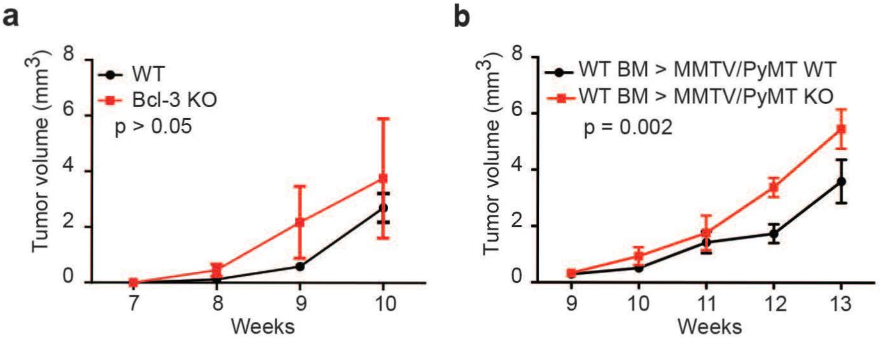

Supplementary Figure 3 Bcl-3 regulates TGFβ signaling by stabilizing Smad3 protein

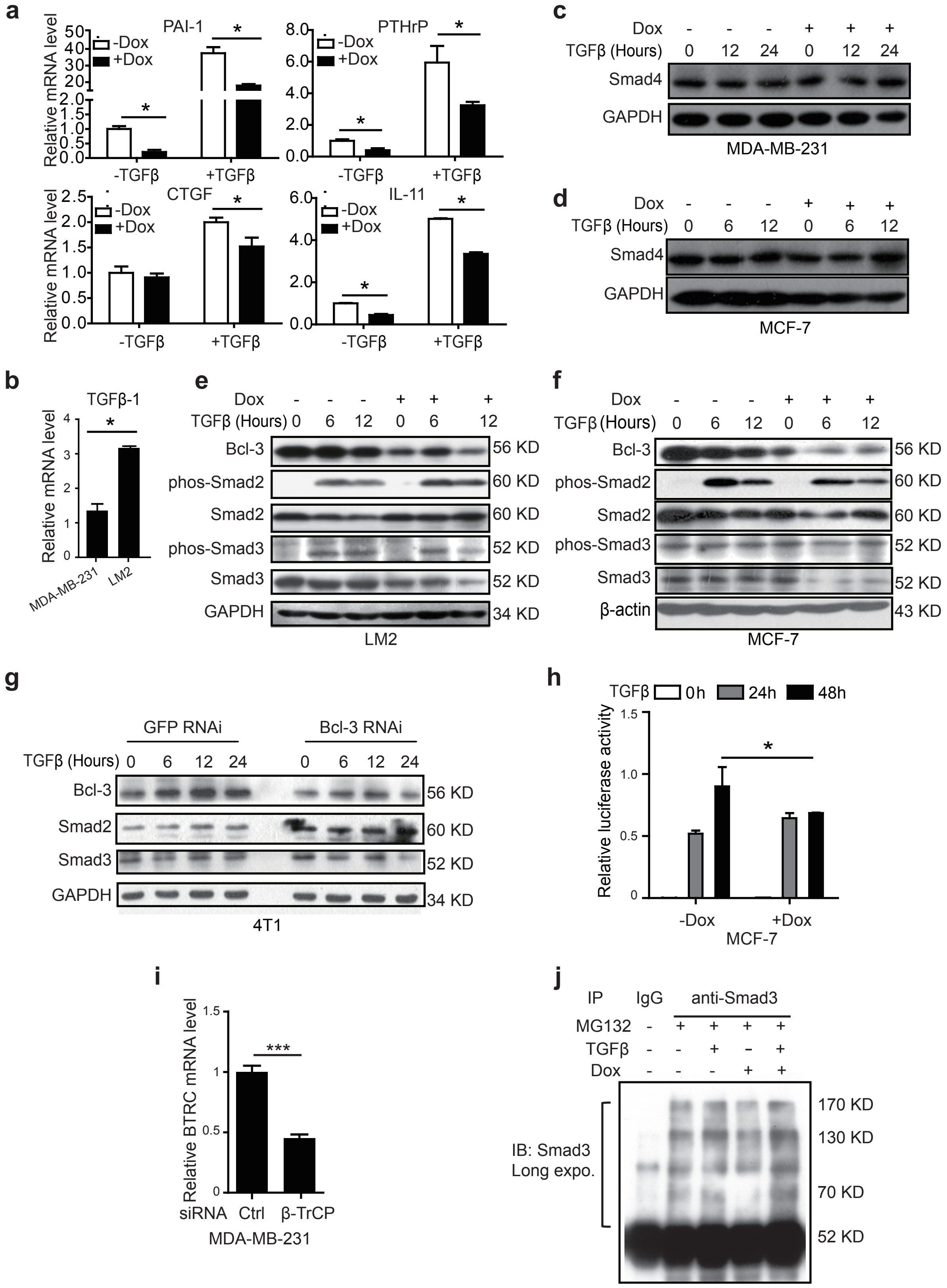

Supplementary Figure 4 Bcl-3 regulation of TGFβ signaling is not correlated with the phosphorylation of Smad3

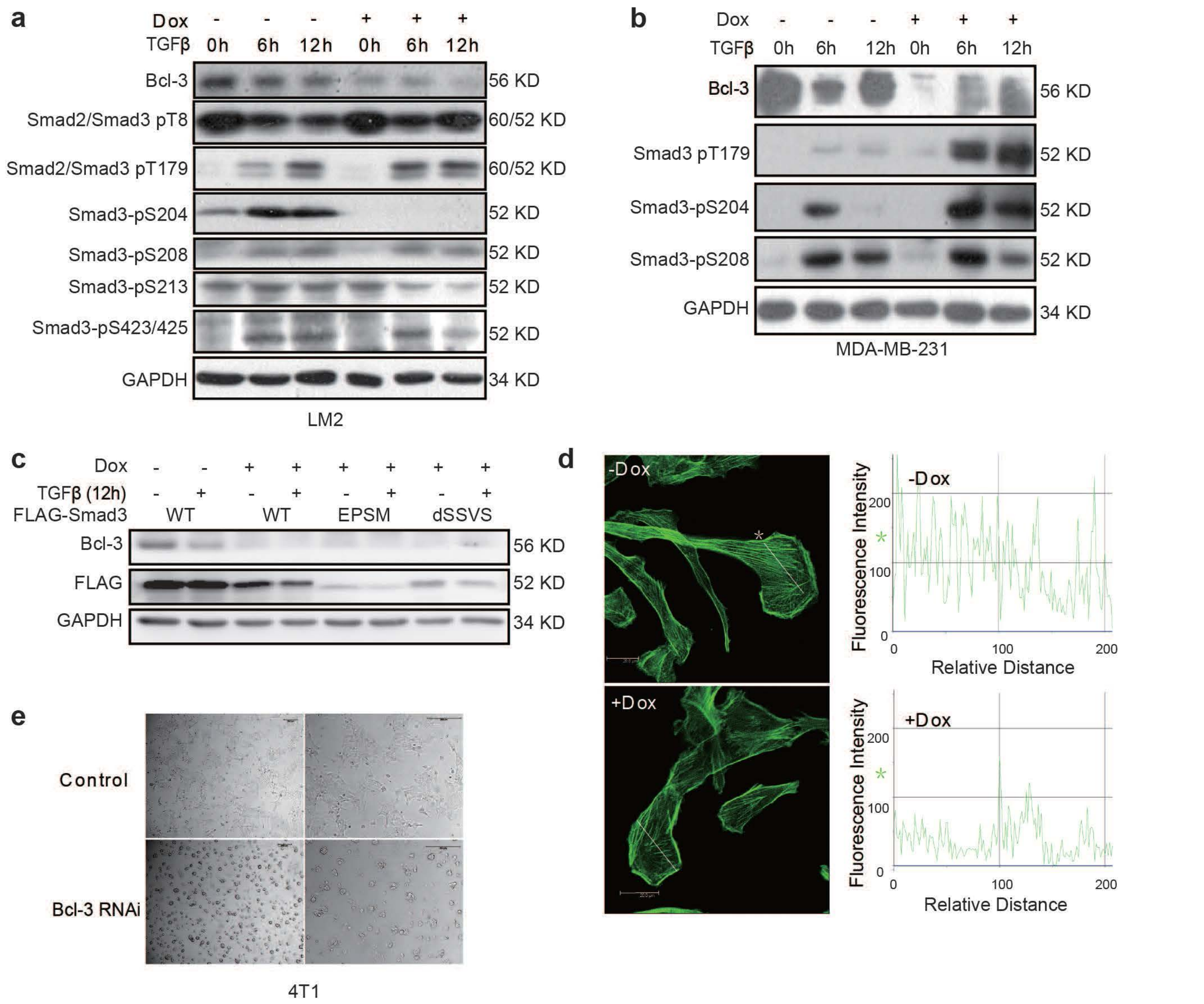

Supplementary Figure 5

Cellular localization of Bcl-3 and Smad3 in TGFβ treated or untreated MDA-MB-231 cells

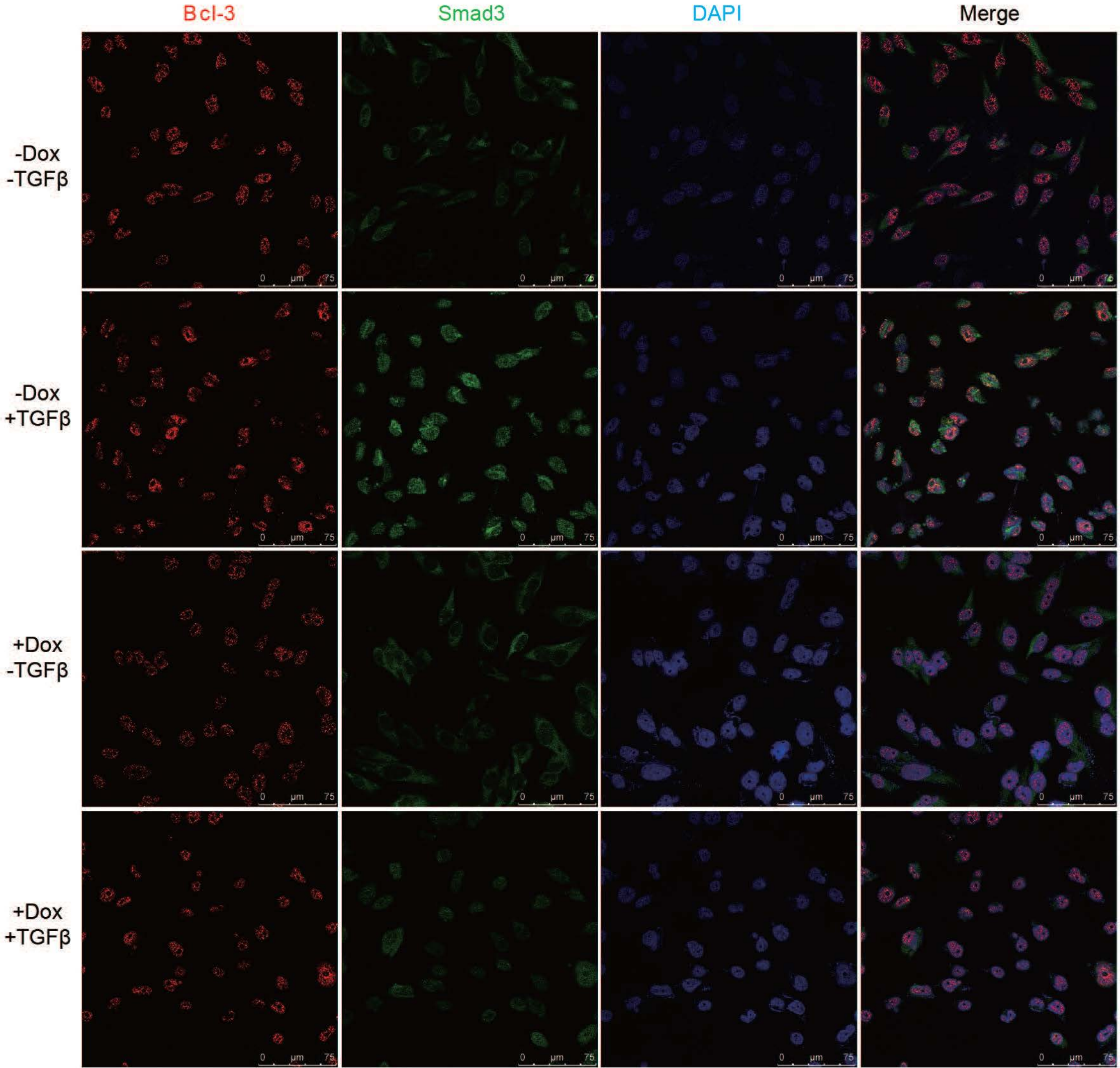

Supplementary Figure 6 Loss of Bcl-3 inhibits the metastasis of breast cancer cells

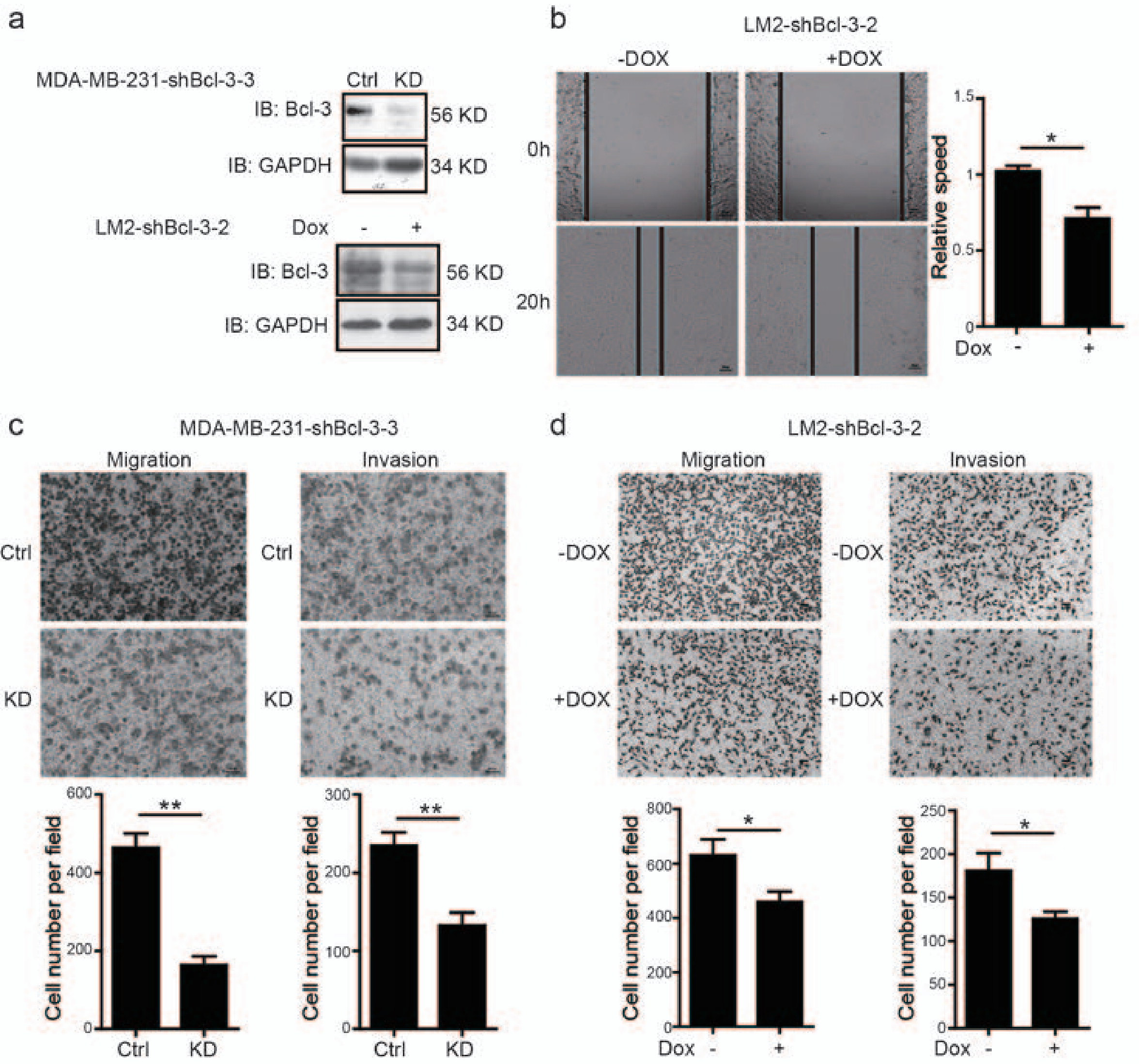

Supplement: Supplementary Figure 1-6 [file cddis2016405x3.pdf]
